# Prevalence and associated factors of early sexual initiation among youth female in sub-Saharan Africa: a multilevel analysis of recent demographic and health surveys

**DOI:** 10.1186/s12905-023-02298-z

**Published:** 2023-03-30

**Authors:** Tigist Andargie Ferede, Atalay Goshu Muluneh, Alemakef Wagnew, Agmasie Damtew Walle

**Affiliations:** 1 Department of Epidemiology and Biostatistics, Institute of Public Health, College of Medicine and Health Sciences, University of Gondar, Gondar, Ethiopia; 2 Department of Health Informatics, College of Health Sciences, Mettu University, Mettu, Ethiopia

**Keywords:** Early sexual initiation, Multilevel analysis, Youth female and sub-Saharan Africa

## Abstract

**Background:**

Early sexual initiation is a major public health concern globally, specifically in Sub-Saharan African (SSA) countries where reproductive health care services are limited. It is strongly related to increased risk of HIV/AIDS, sexually transmitted diseases, unwanted pregnancy, adverse birth outcomes, and psychosocial problems. However, there is limited evidence on the prevalence and associated factors of early sexual initiation among youth females in SSA.

**Methods:**

A secondary data analysis was employed based on the recent DHSs of sub-Saharan African countries. A total weighted sample of 184,942 youth females was considered for analysis. Given the hierarchical nature of DHS data, a multilevel binary logistic regression model was fitted. The Intra-class Correlation Coefficient (ICC), Median Odds Ratio (MOR), and Likelihood Ratio (LR) test were used to assess the presence of clustering. Four nested models were fitted and the model with the lowest deviance (-2LLR0 was selected as the best-fitted model. Variables with *p*-value < 0.2 in the bivariable multilevel binary logistic regression analysis were considered for the multivariable analysis. In the multivariable multilevel binary logistic regression analysis, the Adjusted Odds Ratio (AOR) with the 95% Confidence Interval (CI) was reported to declare the strength and statistical significance of the association.

**Results:**

The prevalence of early sexual initiation among youth females in sub-Saharan Africa was 46.39% [95%CI: 41.23%, 51.5%] ranging from 16.66% in Rwanda to 71.70% in Liberia. In the final model, having primary level education [AOR = 0.82, 95% CI; 0.79, 0.85], and [AOR = 0.50, 95%CI; 0.48, 0.52], being rural [AOR = 1.05, 95%CI: 1.03, 1.07], having media exposure [AOR = 0.91, 95%CI: 0.89, 0.94], and belonged to a community with high media exposure [AOR = 0.92, 95%CI: 0.89,0.96] were found significantly associated with early sexual initiation.

**Conclusion:**

The prevalence of early sexual initiation among youth females in SSA was high. Educational status, wealth index, residence, media exposure, and community media exposure have a significant association with early sexual initiation. These findings highlight those policymakers and other stakeholders had better give prior attention to empowering women, enhancing household wealth status, and media exposure to increase early sexual in the region.

## Introduction

Early sexual initiation is defined as an experience of first intercourse before 15 years of age [1, 2]. Early sexual activity, particularly in developing nations, has been reported to cause social and public health issues [3, 4]. Early sexual practice at a young age is a global public health issue that is particularly prevalent in low-and middle-income countries such as sub-Saharan African countries [5]. According to a report by the World Health Organization (WHO), the annual number of maternal deaths falls from one in 73 to one in 180 [6]. Low and Middle-i accounts for nearly all maternal mortality, with SSA accounting for roughly 66% and Southern Asia accounting for 22% [6].

Globally, about 20 million unsafe abortions and 68,000 unsafe abortion-related deaths occurred annually, of which adolescent girls account for 14% [6]. Studies conducted in different countries evidenced that early sexual initiation increases the risks of unexpected pregnancy and Sexually Transmitted Diseases (STDs). An estimated 30% of teens under the age of 17 have had sexual relations, which is responsible for 252,000 unintended pregnancies per year [7]. According to studies conducted in various parts of the world, the prevalence of early sexual initiation was 9.8% in Malaysia [8], China 18.5% [9] and 58.5% in the Caribbean. A study reported in Brazil showed that the prevalence of early sexual initiation was 7% among girls [10]. Likewise, early sexual initiation is common in African settings ranging from 26% in Nigeria [11] to 55% in Ghana [12].

Early sexual initiation is a significant risk factor for STDs [13]. A girl’s first sexual encounter is frequently unplanned, putting her at risk for STDs, HIV infection, and unwanted pregnancy [7]. Adolescents who have multiple partners early and unprotected stand a high risk of developing HIV and other sexually transmitted diseases, as well as a high prevalence of teenage pregnancy [14, 15]. Mistimed pregnancy and insecure abortion, getting a fistula, and contracting sexually transmitted infections are all important public health concerns in low-income nations today [16].

Given that sexually active young women are at risk of a variety of negative health consequences, including ill health, social, and economic consequences for both women and the feature generation [7]. It increases the likelihood of school dropout, poor academic performance, stigma, and discrimination, as well as STDs like HIV/AIDS, risky sexual practices, unwanted pregnancy, mental illness, and maternal death [17]. It also affects the social and economic position of adults [18].

There is a lack of evidence on the magnitude of early sexual initiation and associated factors in Sub-Saharan Africa. Therefore, this study aimed to assess the Prevalence and associated factors of early sexual initiation among youth females in sub-Saharan Africa. The results of this study could guide public health interventions and programs to reduce the magnitude of early sexual initiation in SSA, which in turn reduces the incidence of child and maternal morbidity and mortality.

## Methods

### Study design, setting, and period

The data source for this study was the Demographic and Health Survey (DHS) data. It is a cross-sectional study conducted every five-year to generate updated health and health-related information. The information was gathered in partnership with the International Classification of Functioning, Disability, and Health (ICF) and Measure DHS in each country [4]. The research was based on the most recent standard Demographic and Health Survey (DHS) of 33 sub-Saharan African countries conducted from 2011–2021. These countries were divided into four regions: Eastern Africa (Burundi, Comoros, Ethiopia, Kenya, Malawi, Mozambique, Rwanda, Tanzania, Uganda, Zambia, Zimbabwe), Central Africa (Angola, Cameroon, Chad, the Democratic Republic of the Congo, Republic of the Congo, Gabon), Western Africa (Benin, Ivory Coast, Gambia, Ghana, Guinea, Mauritania, Liberia) they cover 9.4 million square miles and a total population of 1.1 billion inhabitants. The datasets are found publicly on the DHS website, www.dhsprogram.com. A multi-stage stratified cluster sampling technique was employed to recruit study participants for the survey. The surveys are population-based and have huge sample sizes, and they are nationally representative of each country. A multi-stage cluster sampling procedure was employed in all surveys [12].

### Source and study population

All youth females (aged 15–24 years) in SSA were the source population. The study population was youth females in sub-Saharan African (SSA) countries in the selected Enumeration Areas (EAs).

### Sample size and sampling procedure

In general, all selected national surveys used the most recent census frame. DHS samples are often classified by geographic region or provinces and within each region, by urban/rural areas. The majority of DHS sample designs are a multi-stage sampling technique based on an existing census frame. Enumeration Areas (EAs) were the primary sampling units and Households (HHs) were the secondary sampling unit. Following the listing of households, equal probability systematic sampling is used to select a specified number of households in the designated cluster [12]. Each DHS report on the Measure DHS website included a comprehensive sampling technique (www.dhsprogram.com). Weighted values were computed using Individual women's records (IR) DHS datasets to restore the representativeness of the sample data. Finally, this study comprised a total weighted sample of 184,942 youth females from all 33 nations. A total of 47 countries are located in Sub-Saharan Africa. Of these countries, only 41 countries had a Demographic and Health Survey Report. From these, after excluding countries that had no DHS report after 2011 and countries where the DHS dataset was not publicly available, 33 countries were included in this study (Table 1).

**Table 1:** Sample size determination of early sexual initiation among youth females in each Sub-Saharan Africa: based on 2011–2021 DHS

Regions	Country	survey year	Un Weighted	Weighted Sample size
East Africa Countries	Burundi	2016/17	7,210	7,103
	Comoros	2012	2,284	2,311
	Ethiopia	2016	6,401	6,143
	Kenya	2014	11,483	11,555
	Malawi	2015/16	10,367	10,422
	Mozambique	2011	5,533	5,515
	Rwanda	2019/20	5,732	5,672
	Tanzania	2015/16	5,399	5,387
	Uganda	2016	8,058	8,086
	Zambia	2013/14	6,726	6,631
	Zimbabwe	2015	3,938	3,895
	Angola	2015/16	6,423	6,492
	Cameroon	2018	5,812	5,726
Central Africa countries	Chad	2014/15	6,884	6,992
	DR Congo	2017/18	7,661	7,751
	Congo	2011/12	3,963	4,227
	Gabon	2012	3,407	3,421
	Benin	2017/18	6,251	6,273
	Ivory Coast	2011/12	3,984	3,976
	Gambia	2019/20	4,769	4,814
	Ghana	2014	3,327	3,238
West Africa Countries	Guinea	2018	4,267	4,363
	Liberia	2019/20	3,124	2,765
	Mali	2018	4,116	4,000
	Mauritania	2019/21	6,388	6,403
	Niger	2012	3,869	3,822
	Nigeria	2018	15,267	15,282
	Senegal	2019/20	3,612	3,561
	Sierra Leone	2019	6,062	6,055
	Togo	2013/14	3,337	3,364
Southern Africa Countries	Lesotho	2014	2,842	2,842
	Namibia	2013	3,577	3,691
	South Africa	2016	2,813	8,513
	Total sample size		184,992	184,942

### Outcome variable

The outcome variable of this study was early sexual initiation among youth females which has a binary response (Yes/No). The DHS asked youth females “age at first sexual initiation?”. Then, youth females who had early sexual initiation before the age of 15 coded as “1”, otherwise coded as “0”.

### Independent variables

Independent variables at the individual and community levels were considered. Variables at the individual level were categorized as socio-demographic, pregnancy-related, and behavioral factors. Socio-demographic variables; the age of the youth female, female education, religion; wealth index, marital status, family size, and working status were considered. Characteristics of pregnancy such as parity and pregnancy desirability were also considered. Finally, behavioural characteristics like chewing chat, cigarette smoking, hearing about STIs, and media exposure were included. Media exposure status is created from the frequency of reading a newspaper or magazine, watching TV, and listening to the radio. If a woman has at least one yes, she has considered having media exposure.

Residence, sub-Saharan Africa region, community media exposure, income level of each nation, the survey year, and community women's education were considered as community-level variables. The level of poverty in the community was determined by the proportion of women in the poorer and poorest quintiles obtained from the wealth index results and classified as low (communities in which < 50% of women had poor and poorest wealth quintiles) and high (communities in which ≥ 50% women had poorest and poorer wealth quintiles) poverty communities. Aggregate values measured by the proportion of women with a minimum primary level of education derived from data on respondents' level of education. Then, it was categorized using national median value to values: low (communities with < 50% of women have at least primary education) and high (communities with ≥ 50% of women have at least primary education) community level of women's education. A community-level media exposure is measured by the proportion of women who had been exposed to at least one media; television, radio, or newspaper and classified based on national median value as low (communities with < 50% of women exposed) and high (communities with ≥ 50% of women exposed) [19–21].

### Data management and analysis

This study was conducted using data received from the official DHS measure website www.measuredhs.com. We extracted the outcome and independent variables from the Individual women's Records (IR) data [22]. Based on the guide to DHS Statistics in STATA version 16, data were cleaned and recoded. Before conducting any statistical analysis, we weighted the data for design and non-response using the weighting factor provided in the DHS data, as per the survey report's suggestion, to restore the survey's representativeness and obtain valid statistical estimates.

### Model building

A multi-level binary logistic regression model was fitted to assess factors associated with early sexual initiation. Four models were fitted and the model with the lowest viadeviance (-2LLR) was chosen as the best-fitted model. The first was a null model (Model 1) (a model with no covariates) that fitted to examine the variability of early sexual initiation across the community/EAs. Individual-level variables and community-level variables were included in the second (Model 2) and third (Model 3) models, respectively. In the final model, both individual-level and community-level variables were fitted simultaneously in the fourth model (Model 4). The variance inflation factor (VIF) was used to detect multicollinearity, and all variables had VIF values less than 10 and the mean VIF value of the final model was 1.47.

### Parameter estimation method

Fixed effects (a measure of association) were used to assess the relationship between odds of early sexual initiation among youth females and explanatory variables at both individual and community levels. Variables with a p-value less than 0.2 in the bivariable multilevel binary logistic regression analysis were considered in the multivariable multilevel binary logistic regression analysis. In the multivariable multilevel binary logistic regression analysis, the Adjusted Odds Ratio (AOR) with the 95% Confidence Intervals (CIs) was reported to declare the statistical significance and strength of the association. The random effect measures used to measure the variation were estimated using the Median Odds Ratio (MOR), Intra-class Correlation Coefficient (ICC), and Proportional Change in Variance (PCV). MOR is defined as the central value of the odds ratio between the region's highest risk and the lowest risk when randomly picking out two clusters. The PCV reveals the variation in utilization of early sexual initiation among youth females explained by factors. The ICC, displays the differences in early sexual initiation among youth females between clusters [19, 23, 24].

## Results

### Sociodemographic characteristics of the study population

A total weighted sample of 184,942 youth females was included in this study. Among these youth females, almost half (53.77%) were between the ages of 15 and 19 years. The majority of the research participants (57.57%) lived in rural areas and about 92,366 (49.94%) of the youths attained secondary education. About 94,933 (51.33%) of the youth female belonged to a community with high media exposure and more than half (57.98%) of them were from low-income countries [Table 2].

**Table 2:** Socio-demographic characteristics of youth female in Sub-Saharan Africa: based on 2011–2021 DHS data

Variables	Categories	Weighted frequency	Weighted Percentage
Age of women (in years)	15–19	99,452	53.77
	20–24	84,489	45.23
Maternal educational status	No- education	32,122	17.37
	Primary	60,449	32.69
	Secondary and above	92,366	49.94
Marital status	Not married	132,315	71.5
	Married	52,627	28.46
Religion	Catholic	55,082	29.78
	Muslim	1,955	1.06
	Protestant	93,271	50.43
	Other^a^	34,634	18.73
Wealth index	Poor	64,221	34.72
	Middle	35,764	19.34
	Rich	84,958	45.94
Family size	1–4	62,485	33.79
	5–10	98,293	53.15
	**≥ **11	24,165	13.07
Occupation	No	86,043	46.52
	Yes	98,899	53.48
Heard about STI	No	9,037	4.89
	Yes	175,904	95.11
Media exposure	No	52,247	28.25
	Yes	132,695	71.75
Decision to get medical care	No	38	0.02
	Yes	184,954	99.98
Chewing tobacco	No	156,784	84.77
	Yes	28,158	15.23
Cigarette smoker	No	165,897	89.70
	Yes	19,046	10.30
**Community level variables**			
Place of residence	Urban	78,477	42.43
	Rural	106,465	57.57
Region in SSA	East Africa	72,718	39.32
	Central Africa	34,609	18.71
	West Africa	74,849	40.47
	Southern Africa	2,765	1.50
Income status	Lower	107,236	57.98
	Lower middle	56,016	30.29
	Upper middle	21,690	11.73
DHS year	2011–2015	70,964	38.36
	2016–2021	114,028	61.64
Community media exposure	Low	90,009	48.67
	High	94,933	51.33
Community-women education	Low	92,604	50.06
	High	92,388	49.94

Other^a^ mean religions such as orthodox, Methodist, animist, Jehovah, Universal and no religion, etc.

### The prevalence of early sexual initiation among youth females in SSA

**Fig. 1:**
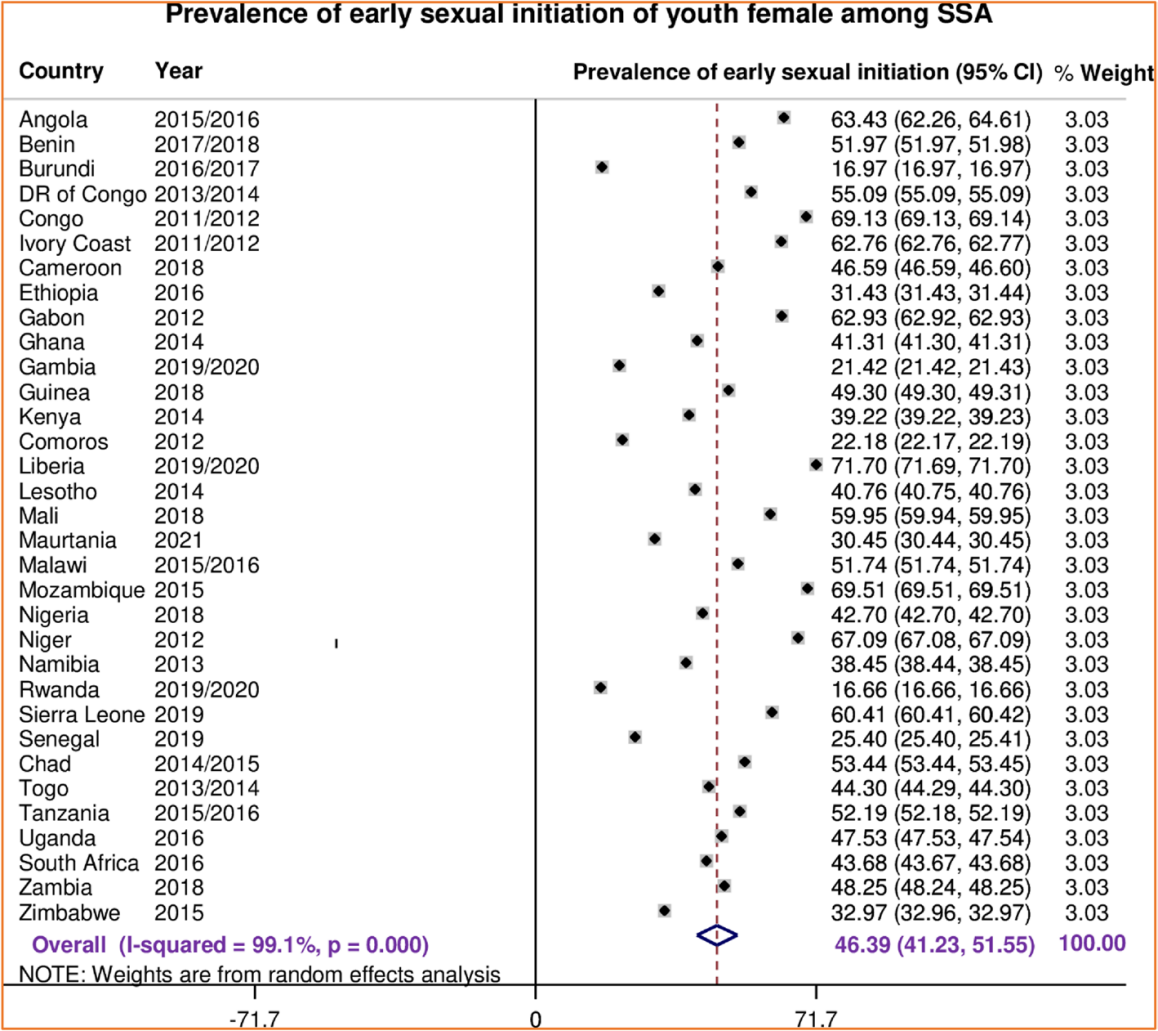
Forest plot of the pooled prevalence of early sexual initiation

The prevalence of early sexual initiation among youth females in sub-Saharan Africa was 46.39% (95% CI; 41.23%, 52.55%) ranging from 16.66% in Rwanda to 71.70% in Liberia (Fig. 1). The prevalence of early sexual initiation varied across sub-Saharan African regions, from the lowest observed in 38.97% (95%CI: 28.91%, 49.03%) in east Africa and highest in 58.43% (95%CI: 52.11%, 64.76.41%) in Central Africa region.

### Model comparison and random effect analysis

As indicated in Table 3, the ICC in the null model was 0.136, which means that about 13.6% of the variations of early sexual initiation among youth females were attributed to the between-cluster variation, but the rest 86.4% were attributed to individual level characteristics. The MOR value was 1.86, which indicates if we randomly choose two youths from different clusters, a youth from a cluster with higher early sexual initiation was 2. 52 times more likely to experience early sexual initiation than youths from a cluster with a lower proportion of early sexual initiation.

**Table 3:** Parameters and model fit statistics for multilevel regression analysis models

**Parameters**	**Null model(**Model 1)	**Model 2**	**Model 3**	**Model 4**
Variance	0.52	0.22	0.16	0.15
ICC	13.6%	6.27%	4.64%	4.36%
MOR	1.86 [1.79,1.93]	1.21	1.03	1.01
PCV	Reff	57.69%	69.23%	71.15%
Model fitness				
Logliklyhood	-126869.89	-113255.68	-123747.31	-102165.82
Deviance	253,739.78	226511.36	247,494.62	204,331.64
Vif	–––––––	1.38	1.19	1.47

*ICC* Inter cluster correlation coefficients*, MOR* Median odds ratio*, PCV* proportional change in variance

*P*-value < 0.05

*P* value < 0.01

*P* value < 0.001

Furthermore, the PCV value in the final model was 71.15% indicating that the variation of early sexual initiation among youth females was explained by the final model. The final model had the lowest deviance value (204,331.64) and was chosen as the best model.

### Multivariable multilevel binary logistic regression analysis

In the final model, individual variables such as female education, marital status, heard about STI, media exposure, wealth index, and occupation, and community-level variables like residence, the region in SSA, income status, and community media exposure were significantly associated with early sexual initiation.

The odds of having early sexual initiation among females who attained primary and secondary or above education were decreased by 18% [AOR = 0.82, 95%CI; 0.79, 0.85] and 50% [AOR = 0.50, 95%CI; 0.48, 0.52] compared to those who had no formal education. The odds of having early sexual initiation among youths who were not married were lowered by 75% [AOR = 0.25; 95%CI; 0.24,0.26] compared to married women. Youth females who had no media exposure had 1.67 times [AOR = 1.67; 95%CI; 1.30,2.15] higher odds of early sexual initiation compared to their counterparts. Women who belonged to middle and rich households had 8% [AOR = 0.92; 95%CI; 0.89, 0.95] and 29% AOR = 0.71; 95%CI; 0.69, 0.73] decreased odds of experiencing early sexual initiation than females belonging to poor households, respectively. The odds of having early sexual initiation among youth females who have worked were 1.32 times [AOR = 1.32; 95%CI; 1.29, 1.35] higher than those who did not work for females. Females who belonged to lower middle and upper middle-income status had 7% [AOR = 0.93; 95%CI; 0.90, 0.95] and 3% [AOR = 0.97; 95%CI; 0.95, 0.99] decreased odds of experiencing early sexual initiation than youth female belonging to poor households respectively. The odds of having early sexual initiation among youth females who heard about STI decreased by 39% [AOR = 0.61; 95%CI; 0.53, 0.70] than youth females who were not heard about it. Having media exposure and belonging to a community with high media exposure decreased the odds of early sexual initiation by 9% [AOR = 0.91; 95%CI; 0.89, 0.94], and 8% [AOR = 0.92; 95%CI 0.89, 0.96], respectively. The odds of having early sexual initiation among youth females who lived in rural residences was 1.05 times [AOR = 1.05; 95%CI; 1.03,1.07] higher than urban residents. Youth females in Central Africa and West Africa were 2.94 [AOR = 2.94; 95%CI; 2.84, 3.05] and 1.61 [AOR = 1.61; 95%CI; 1.56, 1.66] times higher odds of early sexual initiation than a female who lives in East Africa, respectively, however, youth females in Southern Africa were 17% [AOR = 0.83; 95%CI; 0.76, 0.91] decreased odds of early sexual initiation than who live in East Africa [Table 4].

**Table 4:** Multilevel analysis of factors associated with early sexual initiation among youth females in SSA, based on 2011 to 2022 DHS

Variables	Categories	Model 1 AOR [95% CI]	Model 2 AOR [95% CI]	Model 3 AOR [95%CI]	Model 4 AOR [95% CI]
Age of youth female	15–19	––––––	1.00		1.00
	20–24	––––––	1.00[0.98,1.03]	––––––	0.97 [0.95,1.04]
Female education	No- education	––––––	1.00		1.00
	Primary	––––––	0.71[0.68,0.73] **	––––––	0.82[0.79,0.85]**
	Secondary and above	––––––	0.50 [0.49, 0.52] ***	––––––	0.50[0.48,0.52]***
Marital status	Married	––––––	1.00		1.00
	Not Married	––––––	0.27[0.26,0.28] ***	––––––	0.25[0.24,0.26] ***
Wealth index	Poor	––––––	1.00		1.00
	Middle	––––––	0.92[0.89, 0.94] ***	––––––	0.92[0.89,0.95] **
	Rich	––––––	0.68[0.66,0.70] ***	––––––	0.71[0.69,0.73] ***
Religion	Protestant	––––––	1.00		1.00
	Catholic	––––––	0.87 [0.84,0.89] *	––––––	0.97 [0.94,1.00]
	Muslim	––––––	1.02 [0.93,1.11]	––––––	1.04 [0.95,1.13]
	Others	––––––	1.04 [0.96,1.12]	––––––	1.05 [0.98,1.13]
Family size	1–4	––––––	1.00		1.00
	5–10	––––––	0.96 [0.94,0.97] **	––––––	0.98[0.96,1.01]
	** > ** = 11	––––––	1.00 [0.98,1.02]	––––––	1.01[0.99,1.03]
Occupation of female	No	––––––	1.00	––––––	1.00
	Yes	––––––	1.24[1.22, 1.27] ***		1.32[1.29, 1.35] ***
Heard about STI	No	––––––	1.00		1.00
	Yes	––––––	0.43 [0.36,0.50] ***	––––––	0.61[0.53,0.70] ***
Media exposure	No	––––––	1.00		1.00
	Yes	––––––	0.91[0.89,0.94] ***	–––––-	0.91[0.89,0.94] ***
Decision to get medical care	No	––––––	1.00		1.00
	Yes	––––––	0.54[0.29,1.02]	––––––	0.80[0.75,1.66]
Chewing tobacco	No	––––––	1.00		1.00
	Yes	––––––	0.89[0.85,0.93] **	––––––	0.99[0.97,1.01]
Cigar ate smoker	No	––––––	1.00		1.00
	Yes	––––––	1.29[1.22,1.36]	––––––	0.96[0.90,1.01]
**Community level variables**					
Place of residence	Urban	––––––		1.00	1.00
	Rural	––––––	––––––	1.69[1.66,1.73] ***	1.05[1.03,1.07]
Region in SSA	EastAfrica	––––––		1.00	1.00
	Central Africa	––––––	––––––	2.12[2.06,2.18] ***	2.94[2.84,3.05] ***
	West Africa	––––––	––––––	1.61[1.57,1.65] ***	1.61[1.56,1.66] ***
	Southern Africa	––––––	––––––	1.02[0.95,1.11]	0.83[0.76,0.91] **
Income status	Lower	––––––		1.00	1.00
	Lower middle	––––––	––––––	0.84[0.82,0.67] ***	0.93 [0.90,0.95] **
	Upper middle	––––––	––––––	0.95[0.93,0.98] **	0.97[0.95,0.99] ***
DHS year	2011–2015	––––––		1.00	1.00
	2016–2021	––––––	––––––	0.91[0.89,0.94] **	0.99 [0.98,1.01]
Community media usage	Low	––––––		1.00	1.00
	High	––––––	––––––	0.87 [0.83,0.91] ***	0.92[0.89,0.96] ***
Community-youth female education	Low	––––––		1.00	1.00
	High	––––––	––––––	0.92[0.89,0.96] **	0.96[0.93,1.02]

Others mean religions such as orthodox, Methodist, animist, Jehovah, Universal no religion, etc.

*AOR* Adjusted odds ratio, *COR* Crude Odds Ratio, *CI* Confidence Interval

^*^ *P* value < 0.05

^**^p value < 0.01

^***^P value < 0.001

## Discussion

This study aimed to identify the individual and community-level predictors of early sexual initiation among youth females in Sub-Saharan Africa. Based on this, the overall prevalence of early sexual initiation among youth females in -sub-Saharan Africa was 46.39% [95%CI: 41.23%, 51.55%).] which is higher than a study conducted in Indonesia 11% [25], brazil, 7% [10], Taiwan 9.3% [26] and china 18.1% [9]. The variations might be related to factors associated with early sexual initiation of these countries being high-income countries with improved socio-economic status, youth females education, gender equity, and reproductive health youth female are services compared to SSA, which in turn could improve their levels of understanding of the consequences of early sexual initiation [26, 27].

The odds of early sexual initiation among youth females who attained primary and above education level were lower compared to those who didn’t attain formal education. Studies reported in Indonesia [28], Nigeria [29], Ghana [12, 15], and Ethiopia [18, 30]. This could be because youth female's education can result in the corresponding improvement in their level of awareness about reproductive health i.e. about the optimal age for sexual initiation and informed of the consequences of early sexual initiation and related comorbidities which may prevent them from involvement [12, 15]. The odds of having early sexual initiation among youth females who heard about STI were lower than in females who were not heard about it. This was supported by other studies [4, 31]. This might be because youth females have awareness of STI, its causes, and consequences, therefore are less likely to have early sexual initiation. Youth females who were not married had lower odds of having early sexual initiation compared to those who were married. In line with studies reported in Ethiopia [16, 32]. The possible justification could be because early marriage is common practice in many African countries and linked with deeply rooted customs, norms, and values of the community, and they are more likely to have early sexual initiation [31]. An employed youth female had higher odds of early sexual initiation compared with unemployed females which is supported by a study done in sub-Saharan Africa [33]. This may be because youth females are exposed to different risky sexual behaviors at their workplace and this sexual assault leads to early sexual initiation [34]. The odds of having early sexual initiation among youth females belonging to rich and middle household wealth were lower than those of youth females who belonged to poor household. It was in line with the previous studies reported in Africa [2, 11, 18]. It could be economically poor respondents who might be cheated by gifts (either in cash or in-kind) which do initiate them to volunteer for sexual activity take part. In our studies youth females from a community with media exposure and media exposure in the households had lower odds of having early sexual initiation compared to those who didn’t have communities with lower media exposure and media exposure in the households. This was supported by previous studies [2, 35, 36], it could be because media exposure could improve youth females' knowledge, attitude, and practices towards early sexual initiation. In our studies Youth females from rural areas had more likely of early sexual initiation compared to urban females; this finding was consistent with previous studies [16, 37, 38]. This may be due to A possible explanation for this could be low awareness of the community on reproductive health issues and the bad consequence of early marriage for rural adolescents [18]. In our studies youth females from lower-middle and -upper-middle-income statuses were less likely to participate in early sexual than youth from lower-income statuses. This is consistent with the study conducted in Africa [12, 39]. This might be because females from low-income families may participate in earlier sexual relations to obtain money and other benefits; whereas upper and lower-middle-income people have good health-seeking behavior and awareness of lifestyle determinants. Youth females in Central Africa, and West Africa were higher odds of early sexual initiation than women who live in East Africa, however, youth females in Southern Africa were decreased odds of early sexual initiation than those who live in East Africa. This is consistent with the study conducted in Africa These could be ascribed to altering conventional norms as a result of globalization, which causes changes in sociodemographic characteristics such as religion, media exposure, education, and youths people’s socioeconomic situation [40, 41]. This difference could be the reason why young females’ sexual initiation is different across the region.

### Strength and limitation of the study

The strength of this study is the use of advanced statistical models, that consider individual/household and community-level predictors, which also increases the paper's quality. Moreover, the results of the current study, which included 36 nations in sub-Saharan Africa, can be simply generalized to the entire SSA. However, the study has the following limitations. The most important variables such as community attitude, norms, values, knowledge, and attitude towards optimal age for sexual initiation were not considered in this study as these variables were not available in DHS. In addition, the cross-sectional nature of the data does not allow for a cause-effect relationship. The finding may also affect due to the DHS survey year variation. Furthermore, because the majority of the health variables in the DHS are dependent on self-report, the study will be susceptible to recall and social desirability bias.

## Conclusion

The prevalence of early sexual initiation among youth females in Sub-Saharan Africa was high compared to others. Individual-level factors such as female education, marital status of the female, wealth index, heard about STIs, female occupation, and media usage, have a significant association with early sexual initiation. From community-level variables, a region in SSA, residences, community income, and community media usage, were found to be significantly associated with early sexual initiation.

Accordingly, the authors recommend that policymakers and health planners would be better to design programs and plans to increase the youth female's awareness about early sexual initiation through formal/informal education considering early sexual initiation and its health impact on women and also governmental and non-governmental organizations should prioritize modifiable socio-economic determinants and scale up maternal health programs to assist rural and the poorest women.

To have a deeper understanding of the factors, future researchers should take into account maternal health and community knowledge, attitudes, and behavior about the reduction of early sexual initiation by adopting a mixed approach (qualitative and quantitative studies). To decrease maternal complications caused by early sexual initiation in the region, special emphasis on associated factors must also be paid to improving maternal healthcare accessibility, utilization, and quality as well as to reduce early sexual debut and promote healthy sexual relationships among young adolescents.

## Data Availability

The Demographic and Health Survey data can be accessed from the Measure DHS program at www.dhsprogram.com.

## Abbreviations

AOR: Adjusted Odds Ratio
COR: Crude Odds Ratio
DHS: Demographic and Health Survey
EA: Enumeration area
ICC: Intra-class Correlation coefficient
ICF: International Classification Function
MOR: Median Odds Ratio
PCV: Proportion Change in Variance
RH: Reproductive Health
STD: Sexual Transmitted Disease
STI: Sexual Transmitted Illness
SSA: Sub-Saharan Africa
VIF: Variance Inflation Factor
WHO: World Health Organization
